# Textbook outcome in hepato-pancreato-biliary surgery: systematic review

**DOI:** 10.1093/bjsopen/zrac149

**Published:** 2022-11-30

**Authors:** Elise Pretzsch, Dionysios Koliogiannis, Jan Gustav D’Haese, Matthias Ilmer, Markus Otto Guba, Martin Konrad Angele, Jens Werner, Hanno Niess

**Affiliations:** Department of General, Visceral, and Transplant Surgery, Ludwig-Maximilians-University Munich, Munich, Germany; Department of General, Visceral, and Transplant Surgery, Ludwig-Maximilians-University Munich, Munich, Germany; Department of General, Visceral, and Transplant Surgery, Ludwig-Maximilians-University Munich, Munich, Germany; Department of General, Visceral, and Transplant Surgery, Ludwig-Maximilians-University Munich, Munich, Germany; Department of General, Visceral, and Transplant Surgery, Ludwig-Maximilians-University Munich, Munich, Germany; Department of General, Visceral, and Transplant Surgery, Ludwig-Maximilians-University Munich, Munich, Germany; Department of General, Visceral, and Transplant Surgery, Ludwig-Maximilians-University Munich, Munich, Germany; Department of General, Visceral, and Transplant Surgery, Ludwig-Maximilians-University Munich, Munich, Germany

## Abstract

**Background:**

Textbook outcome (TO) is a multidimensional measure reflecting the ideal outcome after surgery. As a benchmarking tool, it provides an objective overview of quality of care. Uniform definitions of TO in hepato-pancreato-biliary (HPB) surgery are missing. This study aimed to provide a definition of TO in HPB surgery and identify obstacles and predictors for achieving it.

**Methods:**

A systematic literature search was conducted using PubMed, Embase, and Cochrane Database according to PRISMA guidelines. Studies published between 1993 and 2021 were retrieved. After selection, two independent reviewers extracted descriptive statistics and derived summary estimates of the occurrence of TO criteria and obstacles for achieving TO using co-occurrence maps.

**Results:**

Overall, 30 studies were included. TO rates ranged between 16–69 per cent. Commonly chosen co-occurring criteria to define TO included ‘no prolonged length of stay (LOS)’, ‘no complications’, ‘no readmission’, and ‘no deaths’. Major obstacles for achieving TO in HPB surgery were prolonged LOS, complications, and readmission. On multivariable analysis, TO predicted better overall and disease-free survival in patients with cancer. Achievement of TO was more likely in dedicated centres and associated with procedural and structural indicators, including high case-mix index and surgical volume.

**Conclusion:**

TO is a useful quality measure to benchmark surgical outcome. Future definitions of TO in HPB surgery should include ‘no prolonged LOS’, ‘no complications’, ‘no readmission’, and ‘no deaths’.

## Teaser

Textbook outcome (TO) reflects the ideal outcome after surgery and is a useful quality measure to benchmark surgical outcome. We are the first to propose a uniform definition of TO in hepato-pancreato-biliary surgery, including ‘no prolonged length of stay’, ‘no complications’, ‘no readmission’, and ‘no mortality’.

## Introduction

Hepato-pancreato-biliary (HPB) surgery frequently involves complex procedures, notably those operations undertaken for malignant disease. Many procedures are prone to complications that influence postoperative course and can have a detrimental effect on outcome. Advances in surgical technique and perioperative care have made surgery safer, leading to increased proclivity to consider patients with more advanced disease or co-morbidities as candidates for surgery^1,2^. In light of these changes, reliable measures that assess patient-centred outcome and overall quality of surgical care are indispensable. Ideally, these measures will help guide treatment decisions and ensure that treatment meets appropriate standards.

Textbook outcome (TO) is a composite measure, originally described in colorectal cancer surgery, that aims to reflects the ideal surgical outcome in a single indicator^3^. TO is achieved when all prespecified parameters are fulfilled according to an all-or-none principle. In this regard, TO can provide a global picture and an overall reflection of surgical quality and care. In turn, identification of parameters that have the greatest influence on TO achievement can help to target single issues and make specific changes accordingly to improve healthcare quality. In this respect, TO has been suggested as a new benchmarking tool to measure internal surgical quality and provide quality-of-care information to patients. Indeed, patients prefer summary measures regarding quality-of-care information^4,5^.

A uniform definition of TO in HPB surgery is presently missing^6–9^. Ideally, included parameters should be disease and surgery specific.

This study aimed to systematically review literature to investigate existing definitions of TO in HPB surgery and provide the basis for a uniform definition of this composite measure. The primary objective was to assess parameters used for defining TO. Secondary objectives were TO achievement rate; identifications of factors influencing TO achievement; relation between TO and minimally invasive surgery, type of resection (minor *versus* major), survival, hospital performance (procedural volume, case-mix index (CMI)), type of hospital, hospital designation (including Magnet status), and socioeconomic factors.

## Methods

### Search strategy

**Fig. 1:**
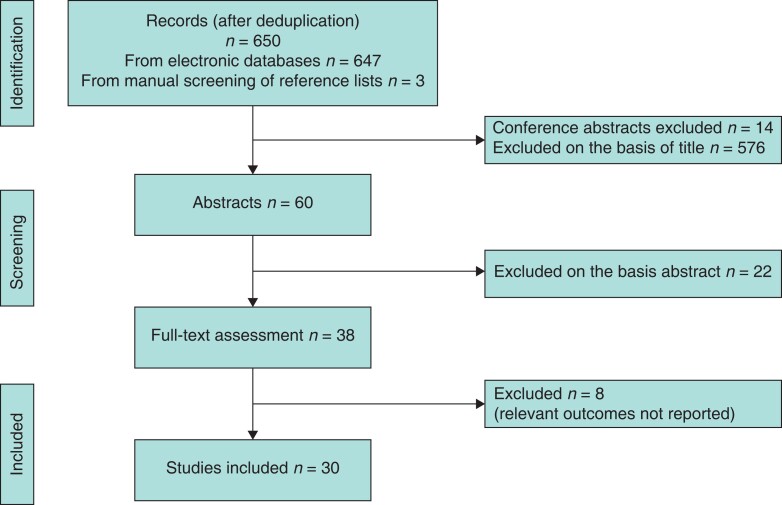
Flow chart of literature selection strategy

A systematic literature search was conducted using PubMed, Embase, and Cochrane Database according to the PRISMA guidelines^10^ by two independent investigators. All studies published in English until December 2021 were potentially eligible for inclusion. The search terms were (textbook outcome) OR (textbook AND outcome*). The reference list of each article was searched for further relevant literature. Duplicate articles, editorials, and conference abstracts were excluded. The articles were screened and filtered by title and abstract. A full-text assessment and review of the remaining studies was conducted. Data from the included articles were extracted independently by the two reviewers using double-data extraction. Inconsistencies were resolved by consensus. In the case of disagreement, a third reviewer was consulted so that consensus was reached (*Fig. 1*).

### Eligibility criteria

All studies reporting TO in patients 18 years or older who underwent either hepatic, biliary, or pancreatic surgery or a combination of these for any condition (benign or malignant) were considered eligible for inclusion. Multivisceral resections including other compartments (for example rectal resection) were excluded.

### Data extraction and analysis

The following data were collected: author details, year of publication, country, recruitment interval, study population, sample size, diagnosis, procedure, definition of TO with all included components (including the percentage of the respective components reached), occurrence of TO, independent predictors of TO, any data with regard to hospital status and performance (procedural volume, CMI^11^, teaching status, and Magnet designation^12^), expenditure, social vulnerability, and racial diversity.

### Data visualization

#### Co-occurrence matrix

**Fig. 2:**
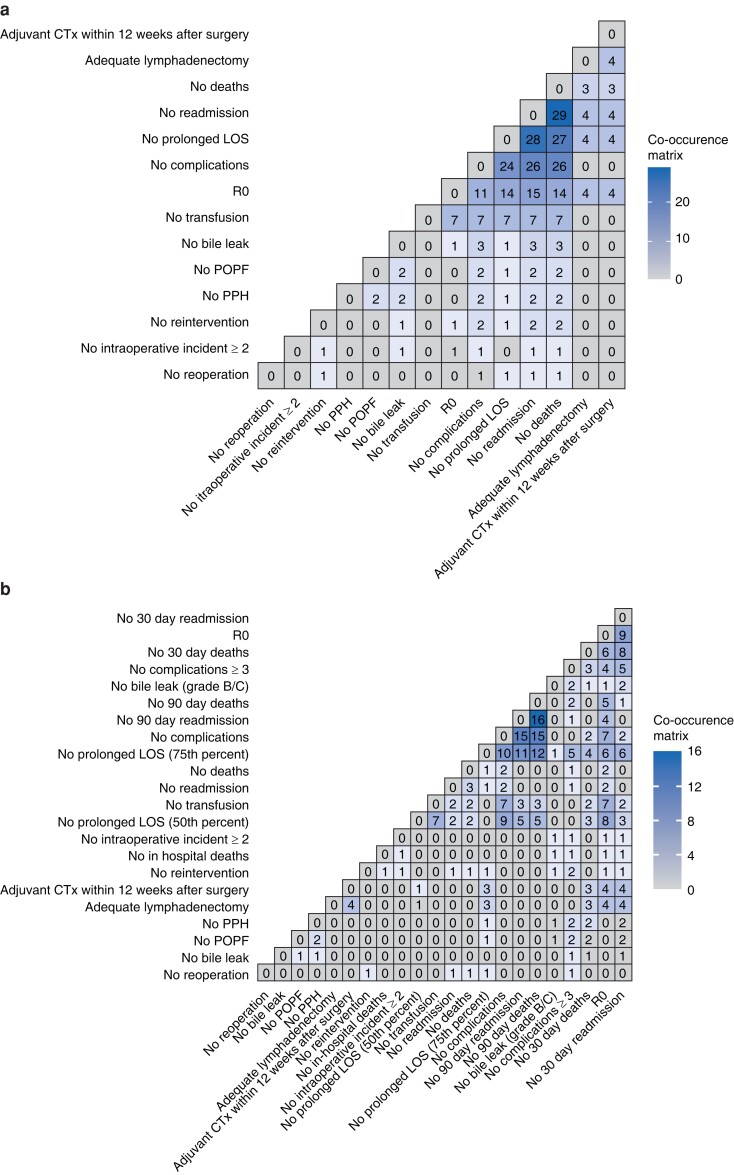

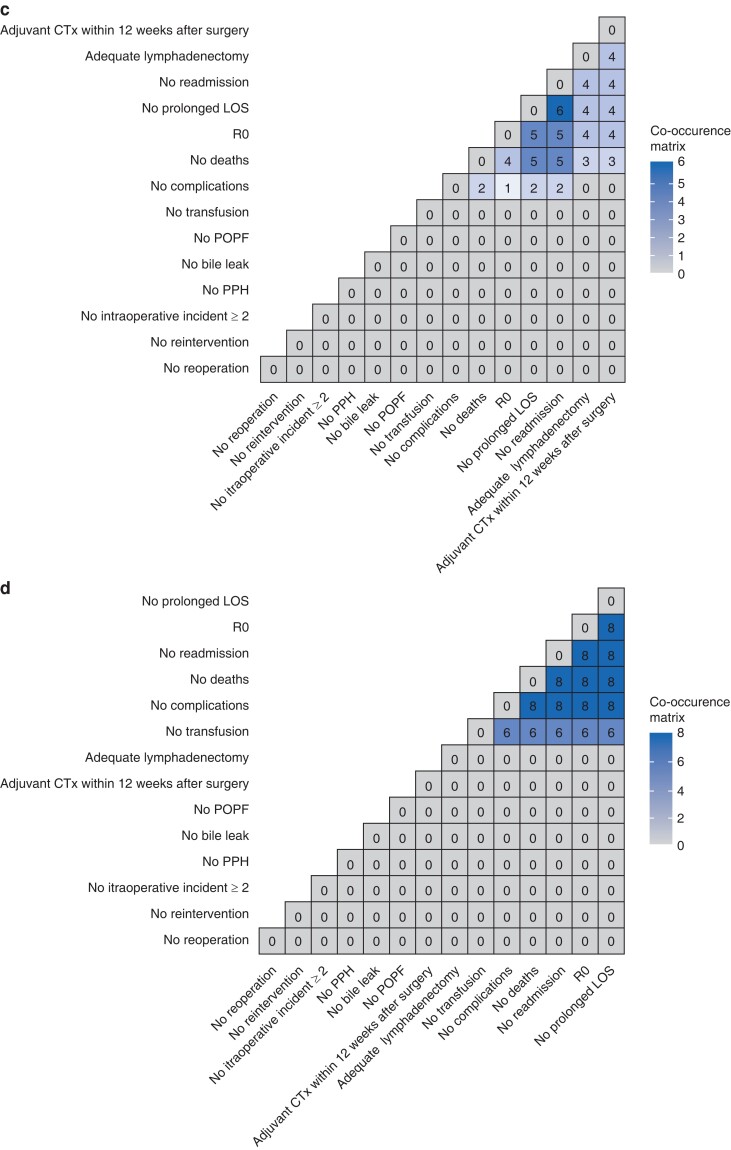
Definition of textbook outcome Co-occurrence maps visualizing how many times each TO criterion was examined in the context of all other criteria across studies. TO criteria are shown along the *x* and *y* axis, with matrix values (and tile colours) indicating the amount of co-occurrence. **a** All HPB studies, similar TO criteria were grouped into summary variables: ‘no prolonged LOS’ (summarizing: no prolonged LOS in either the 50th or 75th percentile), ‘no readmission’ (30 days, 90 days, any), ‘no deaths’ (30 days, 90 days, in-hospital, any), ‘no complications’ (three or more, any), and ‘no bile leak’ (grade B/C, any), **b** All HPB studies, similar TO criteria are all shown individually, **c** Studies focusing on pancreatic cancer only. **d** Studies focusing on hepatobiliary malignancies only. HPB, hepato-pancreato-biliary; TO, textbook outcome; CTx, chemotherapy; LOS, length of stay; POPF, postoperative pancreatic fistula; PPH, post-pancreatectomy haemorrhage.

Co-occurrence maps were computed to visualize how many times each TO criterion was considered. Co-occurrence is defined as an above-chance frequency of occurrence of two terms/criteria^13^. These maps show TO criteria along the *x* and *y* axis, with matrix values (and tile colours) indicating the amount of co-occurrence. Co-occurrence maps were generated first including all studies on hepatobiliary surgery for both benign and malignant disease (*Fig. 2a*) and second, focusing on only subgroup analyses of pancreatic cancer (*Fig. 2c*) and only hepatobiliary malignancies (*Fig. 2d*). Summary graphs were also provided (*Fig. 2b*). Summary variables included ‘no prolonged length of stay (LOS)’ in either the 50th or 75th percentile, ‘no readmission’, and ‘no deaths’ at 30 days, 90 days, in-hospital, and any time, ‘no complications’ either overall or three or more, and ‘no bile leak’ grade B/C, or any as defined by the International Study Group for Liver Surgery^14^.

### TO and specific parameter achievement rates

Rates of patients that achieved each TO criterion were derived from the original publication or, where not available, computed by the authors of this study. In the presence of subgroups, averages were weighted by the number of participants within each subgroup.

#### Multivariable contributors

To help readers visualize the contribution of each parameter to TO achievement, negative (indicated in blue) and positive (indicated in red) contributors were displayed using word clouds. The size of each word/term reflects the strength of its contribution to TO. Where identical terms were present across multiple studies, their OR values were averaged. Studies with missing values were excluded. To indicate the relevance of each contributor to achieving TO in the context of a particular diagnosis, diagnostic labels in parentheses following terms (where applicable) were added.

## Results

### Literature search

A total of 650 studies were identified after de-duplication. Of these, 14 conference abstracts were excluded. Screening by title led to the exclusion of 576 studies, 22 studies were excluded after abstract review, leaving 38 studies that underwent full-text assessment, with 30 meeting eligibility criteria (*Fig. 1*). The eight studies excluded did not reported the relevant outcomes.

Overall, 10 studies used administrative data from Medicare Inpatient and Outpatient Standard Analytic Files with in-part overlapping cohorts^12,15–23^. As they were focused on different quality metrics, they were all included in a purely descriptive manner to give the broadest overview on the current knowledge on TO.

### Study characteristics

The characteristics of the included studies are shown in *Table 1*. All studies retrieved information from prospectively maintained databases. Study intervals ranged from 1993 to 2020. Two studies started before 2000, 12 studies before 2010, and the remaining 16 studies from 2010 onwards. More than half of the included studies were from the USA (16), six were international, and five were from France. The remaining studies were from UK (one), The Netherlands (one), Spain, and France (one binational study). There were 28 multi-institutional studies. The sample size of patients per study ranged from 78 to 42 551. Ten studies had a sample size lower than 1000, six between 1000–10 000, and 14 more than 10 000.

**Table 1:** Characteristics of the included studies

Author	Diagnosis	Procedure	*n*	Recruitment interval	Overall TO rate (%)	TO rate (%) subgroups
**Pancreatic surgery**						
Aquina *et al*.^24^	Malignancy	Pancreatic surgery	42 551	2006–2017	25	
Beane *et al*.^25^	Any indication	Pancreatic surgery	24 168	2013–2017	NA	PD: 55.4 DP: 55.8
Diaz *et al*.^18^	Any indication	Pancreatic surgery	24 298	2013–2017	43.3	Low diversity area: 40 Average diversity area: 43 High diversity area: 49
Heidsma *et al*.^26^	PNET	Pancreatic surgery	821	2000–2016	49.3	PD: 32.5 DP: 56.7 enucleation: 52
Kulshrestha *et al*.^27^	PDAC	PD	16 602	2006–2015	21.5	high volume: 26.2 moderate volume: 18.5 low volume: 17.3 very low volume: 12.2
Lof *et al*.^28^	Any indication	PD (only open)	375	2009–2017	NA	ERAS: 56.4 Non-ERAS: 44
Mehta *et al*.^21^	Malignancy	Pancreatic surgery	26 268	2016–2017	NA	Trifactor status: 49.7 Non-trifactor status: 42.9
Merath *et al*.^29^	Any indication	Pancreatic surgery	4853	2013–2015	NA	
Sweigert *et al*.^6^	PDAC	PD	18 608	2006–2016	16.8	Over time, in 2007: 12.9 Over time, in 2015: 19.5
Sweigert *et al*.^30^	PDAC	PD	12 854	2010–2015	NA	Laparoscopic: 24.7 Open: 23.5
Van Roessel *et al*^8^	Any indication	Pancreatic surgery	3341	2014–2017	60.3	PD: 58.3 DP: 67.4
**Liver surgery**						
Azoulay *et al*.^31^	HCC (CSPH ≥ 10mmHg)	Liver surgery	79	1999–2019	34	
Brustia *et al*.^32^	ICC	Liver surgery	855	2000–2018	15.8	Laparoscopic: 30.8 Open: 12.7
Görgec *et al*.^9^	Any indication	Liver surgery	8188	2011–2019	69.1	Laparoscopic: 74.8 Open: 61.9
Hobeika *et al*.^33^	HCC	Liver surgery	425	2010–2018	32.9	Laparoscopic: 38.7 Open: 24.2
Hobeika *et al*.^34^	Any indication	Liver surgery (only laparoscopic)	1343	2000–2017	NA	left lateral sectionectomy: 43.7 right hepatectomy: 23.8
Hobeika *et al*.^35^	ICC	Liver surgery	548	2000–2017	22.1	Laparoscopic: 28.3 Open: 20.2
Merath *et al*.^36^	ICC	Liver surgery	687	1993–2015	25.5	Eastern countries: 25.7 Western countries: 66.7
Nassar *et al*.^37^	Malignancy	Liver surgery (only laparoscopic)	463	2009–2019	NA	Difficulty level A: 39.1 Difficulty level B: 36.8 Difficulty level C: 30.8
Paro *et al*.^23^	Any indication	Liver surgery	13 898	2013–2017	53.2	MetS: 46.8 No MetS: 54.6
Tsilimigras *et al*.^38^	HCC	Liver surgery	605	2000–2015	62.3	
Tsilimigras *et al*.^39^	HCC, ICC	Liver surgery	1829	2005–2017	62	HCC: 68 ICC: 55
Yoshino *et al*.^40^	Any indication	Liver surgery	78	2000–2020	38	
**Hepatopancreatic surgery**						
Azap *et al*.^17^	Any indication	Hepatopancreatic surgery	32 142	2013–2017	51.2	Resection pancreas: 46.6 Resection liver: 57.6 Low SVI: 52.8 Intermediate SVI: 51.5 High SVI: 49.8
Hyer *et al*.^11^	Any indication	Hepatopancreatic surgery	31 452	2013–2017	50.7	Low CMI: 45.7 Average CMI: 47.5 High CMI: 53
Mehta *et al*.^20^	Malignancy	Hepatopancreatic surgery	35 352	2013–2015	NA	Resection pancreas: 37.8 Resection liver: 38.5
Mehta *et al*.^19^	Malignancy	Hepatopancreatic surgery	8035	2013–2015	44.3	Minor teaching status: 40 Major teaching status: 45.4
Mehta *et al*.^22^	Malignancy	Hepatopancreatic surgery	21 234	2013–2017	45.6	
Mehta *et al*.^12^	Malignancy	Hepatopancreatic surgery	10 997	2015–2017	45.6	Magnet status: 46 Non-Magnet status: 45
Merath *et al*.^15^	Any indication	Hepatopancreatic surgery	13 467	2013–2015	44	Minor resection pancreas: 47.8 Major resection pancreas: 24.7 Minor resection liver: 46.8 Major resection liver: 33.3

CMI, case-mix index; CSPH, clinically significant portal hypertension; DP, distal pancreatectomy; ERAS, enhanced recovery after surgery; HCC, hepatocellular carcinoma; ICC, intrahepatic cholangiocellular carcinoma; MetS, metabolic syndrome; NA, not available; PD, pancreatoduodenectomy; PNET, pancreatic neuroendocrine tumour; SVI, social-vulnerability index.

### Patient characteristics

Most studies were focused on patients receiving HPB surgery for a malignant indication (*n* = 18). Among those, six included only patients with pancreatic ductal adenocarcinoma (PDAC), eight focused on liver cancer (three hepatocellular carcinoma (HCC), three intrahepatic cholangiocarcinoma (ICC), one HCC and ICC, and one primary and secondary malignancy), and four looked at HPB malignancies as a whole.

### Definition of textbook outcome

Parameters used to define TO are shown in *Fig. 2*. The most frequently used and co-occurring measure were ‘no prolonged LOS’, ‘no readmission’, ‘no deaths’, and ‘no complications’ (*Fig. 2a*). Multiple definition existed for these parameters. ‘No prolonged LOS higher or equal to the 75th percentile’, ‘no readmission within 90 days’, ‘no deaths within 90 days’, and ‘no complications’ were the most used (*Fig. 2b*). *Figure 2c,d* shows the results for subgroup analysis with regard to PDAC (*Fig. 2c*) and hepatobiliary malignancies (*Fig. 2d*).

### Achievement of textbook outcome

The rate of patients achieving TO ranged between 15.8–69.1 per cent, 16.8–60.3 per cent, and 15.8–69.1 per cent in all candidates, pancreatic surgery candidates (including benign and malignant disease), and liver surgery candidates (including benign and malignant disease) respectively. Median TO rate was 38 per cent. Patients with malignant disease presented lower TO rates than the median and patients with benign disease. Patients with benign disease had a TO rate higher than the median. (*Table 1*).

### Major obstacles in achieving textbook outcome

**Fig. 3:**
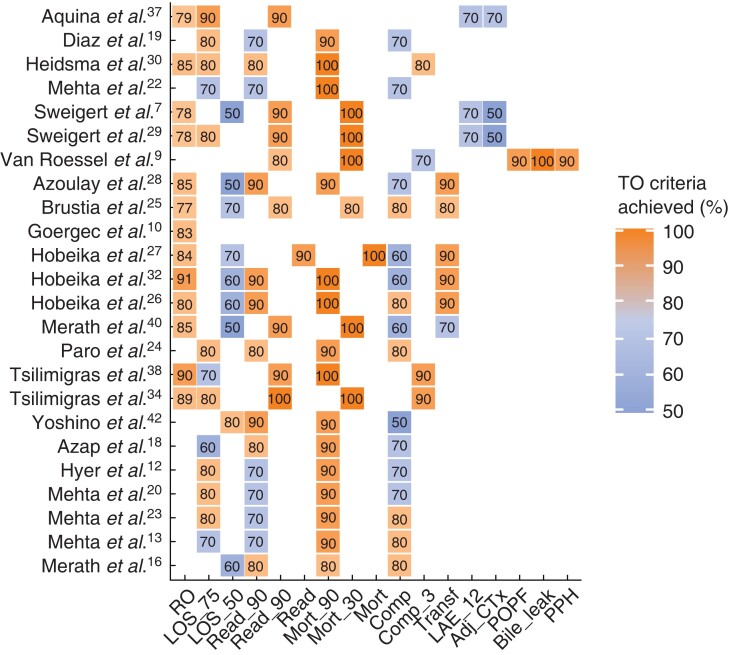
Components of textbook outcome and percentages reached Overview of criteria chosen to define TO within each study with percentages of patients reaching each component (studies with missing data are not included). The colour gradient highlights that the major obstacles for achieving TO across all studies were ‘prolonged LOS’, ‘complications’, and ‘readmission’. LOS, length of stay; read, readmission; mort, deaths; comp, complication; transf, transfusion; LAE, lymphadenectomy; adj CTx, adjuvant chemotherapy; POPF, postoperative pancreatic fistula; PPH, post-pancreatectomy haemorrhage; TO, textbook outcome.

*Figure 3* gives an overview of the parameters chosen to define TO within each study, including the percentage of patients reaching each component. Accordingly, the major obstacles for achieving TO across all studies were ‘prolonged LOS’, ‘complications’, and ‘readmission’.

### Textbook outcome and operative approach

Seven studies investigated the TO rate with regard to the operating approach. All studies found better TO rates in patients undergoing a laparoscopic procedure compared with an open procedure. Two of these studies included only patients with ICC^32,35^ and three studies compared the laparoscopic and open approach in patients with HCC, portal hypertension and cirrhosis, and patients with any liver disease^9,31,33^. In both studies focusing on HCC, the laparoscopic approach proved to be a predictor of TO in multivariable analysis with Ors of 2.81 (95 per cent c.i. 1.29 to 6.12) and 5.6 (95 per cent c.i. 1.7 to 18.2)^31,33^. One study investigating patients who underwent HPB surgery for any indication reported that a laparoscopic approach was an independent predictor of TO achievement with an OR of 1.52 (95 per cent c.i. 1.34 to 1.73)^15^. One study investigating TO with respect to operating approach among patients with PDAC failed to identify a difference in TO rates between minimally invasive and open pancreaticoduodenectomy^30^.

### Textbook outcome, type, and extent of operation

TO ranged between 32.5–58.3 per cent, and 55.8–67.4 per cent after pancreaticoduodenectomy and distal pancreatectomy respectively^8,25,26^. PDAC (OR 1.36, 95 per cent c.i. 1.14 to 1.63) and a dilated pancreatic duct (more than 3 mm) (OR 2.22, 95 per cent c.i. 2.05 to 3.57) were identified as independent prognostic factors of TO achievement after pancreatoduodenectomy (OR 1 or higher) in one study^8^.

Among hepatobiliary procedures, previous liver resection, ICC, gallbladder carcinoma, increasing tumour size (more than 3 cm), minor resection of posterior/superior segments, non-anatomical resection, (anatomical) major resection, biliary reconstruction, and major vascular invasion were all associated with decreased likelihood of achieving TO (OR 1 or less)^9,15,23,33,39^.

### Textbook outcome, hospital status, and performance

#### Case-mix index

Hospital CMI was strongly associated with the probability of achieving TO in one study. TO rate was 45.7 per cent and 53 per cent in facilities with low and high CMI respectively. A low as well as an average CMI were significantly associated with lower odds of achieving TO (OR 0.78, 95 per cent c.i. 0.69 to 0.87 and OR 0.82, 95 per cent c.i. 0.76 to 0.88 respectively) when compared with high CMI. Even after adjusting for HPB surgical volume at the hospital level, CMI had a beneficial effect on the odds of achieving TO^11^.

#### Surgical volume

Twelve studies evaluated the relationship between TO and surgical volume. Seven studies reported a significant positive correlation, whereas five did not (*Table 2*).

**Table 2:** Textbook outcome and surgical volume

Author	Diagnosis	Specific volume parameters assessed	OR (95% c.i.)	*P*
**Multivariate analysis**				
Kulshrestha *et al*.^27^	Pancreatic cancer	High volume (*versus* very low)	2.39 (2.02–2.85)	*P* < 0.001
Mehta *et al*.^12^	Hepatopancreatic malignancies	Magnet centre: Leapfrog volume compliant (*versus* noncompliant), Non-Magnet centre: Leapfrog volume compliant (*versus* noncompliant)	1.24 (1.06–1.44), 1.18 (1.11–1.26)	*P* < 0.001
Hobeika *et al*.^34^	Any liver disease	In Laparoscopic right hepatectomies: ≥ 35 liver resections/year, In laparoscopic left lateral sectionectomies: ≥ 25 liver resections/year	2.55 (1.34–5.63), 2.45 (1.65–3.69)	*P* < 0.001
Mehta *et al*.^19^	Hepatopancreatic malignancies	High volume (*versus* low)	1.30 (1.15–1.47), 1.19 (1.03–1.38)	*P* < 0.001
Sweigert *et al*.^6^	*P*ancreatic cancer	Low volume (<20 PD/year) (*versus* high)	0.49 (0.38–0.64)	*P* < 0.001
Merath *et al*.^29^	Any pancreatic disease	Leapfrog volume compliant (*versus* noncompliant)	1.28 (1.09–1.50)	*P* < 0.001
**Univariate analysis**				
Azap *et al*.^17^	Any hepatopancreatic disease	High volume (*versus* low)		*P* < 0.001
Merath *et al*.^15^	Any hepatopancreatic disease	Not further specified		*P* > 0.050
Tsilimigras *et al*.^38^	HCC	Not further specified		*P* > 0.050
Van Roessel *et al*.^8^	Any pancreatic disease	Not further specified		*P* > 0.050
Görgec *et al*.^9^	Any liver disease	Not further specified		*P* > 0.050
Merath *et al*.^36^	ICC	Not further specified		*P* > 0.050

HCC, hepatocellular carcinoma; ICC, intrahepatic cholangiocellular carcinoma.

#### Hospital designation

Four studies investigated the relationship between hospital designation and TO. Two studies showed that hospitals meeting the quality trifactor (Leapfrog minimum volume standards, Hospital Safety Grades, and Magnet recognition) had higher odds of TO (OR 1.37, 95 per cent c.i. 1.21 to 1.55 and OR 1.28, 95 per cent c.i. 1.03 to 1.59) and lower odds of complications, prolonged LOS, and deaths^21,29^.

One study suggested that dedicated cancer centres provide higher-value surgical care for patients with HPB malignancies. Patients treated in dedicated cancer centres, even when presenting with a higher co-morbidity burden, had higher odds of achieving TO (pancreatic surgery OR 1.71, 95 per cent c.i. 1.50 to 1.95; liver surgery OR 1.36, 95 per cent c.i. 1.15 to 1.62) compared with those treated in other hospitals^22^.

#### Socioeconomic and segregation factors

Patients with a high social-vulnerability index (SVI) were less likely to achieve TO after HPB surgery (pancreatic surgery OR 0.89, 95 per cent c.i. 0.82 to 0.97; and liver surgery OR 0.89, 95 per cent c.i. 0.80 to 0.98) in one study. A high SVI was an independent predictor of complications and 90-day deaths after both pancreatic and liver surgery as well as prolonged LOS in liver surgery^17^. Socioeconomic factors independently associated with achievement of TO were lower patient education, government insurance, low county-level racial diversity, and Black race^6,18,24,27^.

#### Expenditure

Four studies investigated the relationship between expenditure (insurance payments) and TO. All reported that payments for patients achieving TO were markedly lower (about $10 000 less) than payments for patients who did not achieve TO^15,17,19,22,23^.

### Textbook outcome and survival

Eleven studies assessed the impact of TO on survival. While two studies did not report better survival rates when TO was achieved^32,35^, the remaining nine studies did ^6,24,26,27,30,31,33,38,39^ (*Table S1*).

Tsilimigras *et al*. reported that among patients with HCC who achieved TO, 5-year overall survival (OS) was higher (69.6 per cent *versus* 56.9 per cent) compared with patients who did not. TO achievement was associated with 40 per cent decreased risk of death with respect to all-cause deaths (HR 0.60, 95 per cent c.i. 0.42 to 0.85)^38^. Another study reported that TO was independently associated with 26 per cent and 37 per cent decreased hazards of death among ICC (HR 0.74, 95 per cent c.i. 0.56 to 0.97) and patients with HCC (HR 0.63, 95 per cent c.i. 0.46 to 0.85) respectively^39^. Similarly, in a study by Sweigert *et al*. on patients with PDAC, TO achievement was associated with better OS (median 27 *versus* 19.9 months) and lower risk of long-term deaths (HR 0.73, 95 per cent c.i. 0.70 to 0.77)^6^. Three studies on PDAC concluded that TO was associated with better OS (TO median, 26.7 months *versus* no TO median, 21.1 months)^30^, a 4.8-month increase in median survival at any facility^27^, and that all components of TO (in this study: R0, adequate lymphadenectomy, no prolonged LOS, no readmission, and start of adjuvant chemotherapy within 12 weeks after surgery) were independently associated with the 5-year OS^24^. Ultimately, two studies investigating the impact of TO on disease-free survival (DFS) in patients with HCC and pancreatic neuroendocrine tumours reported that TO was independently associated with DFS (HR 0.34, 95 per cent c.i. 0.19 to 0.60 and HR 0.49, 95 per cent c.i. 0.32 to 0.75 respectively)^26,33^.

### Textbook outcome, patient characteristics, and perioperative factors

**Fig. 4:**
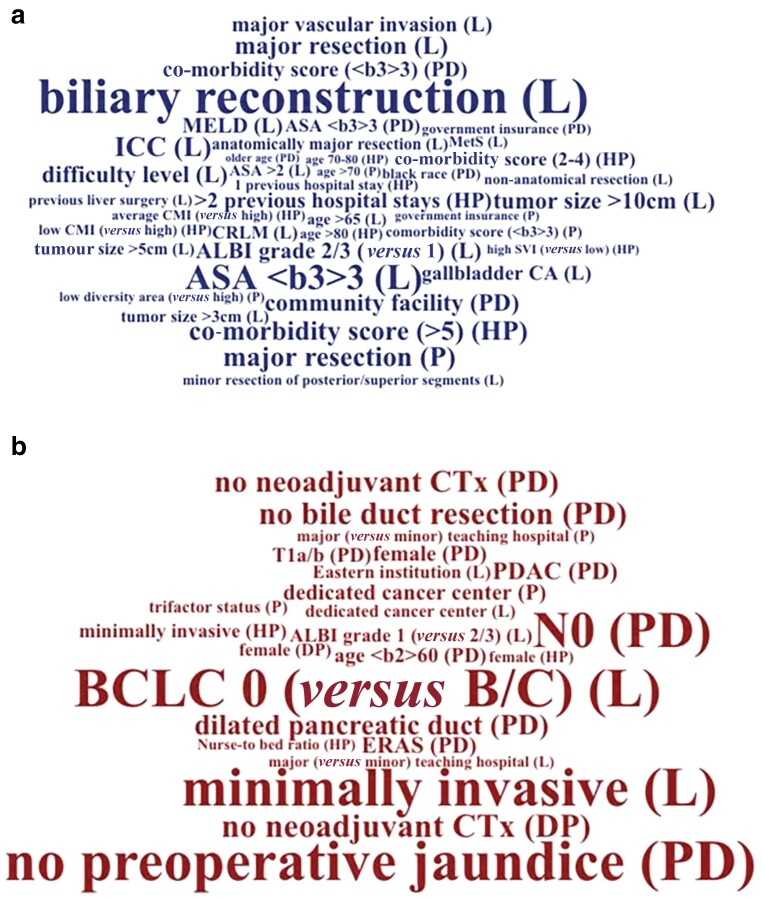
Multivariate contributors to textbook outcome For ease of interpretation, negative (OR less than 1), indicated in blue, **a** and positive (OR higher than 1, indicated in red, **b** contributors are visualized using word clouds. The size of each word/term reflects the strength of its (negative/positive) contribution to TO. Specifically, weak and strong contributions are represented by small and large font. To indicate the relevance of each contributor to achieving TO in the context of a particular diagnosis, we added diagnostic labels in parentheses following terms (where applicable). L, hepatic surgery; HP, hepatobiliary surgery; PD, pancreatoduodenectomy; TO, textbook outcome; ALBI, albumin-bilirubin; ICC, intrahepatic cholangiocarcinoma; MELD, Model for End-Stage Liver Disease; CMI, case-mix index; SVI, social-vulnerability index; CTx, chemotherapy; BCLC, Barcelona Clinic Liver Cancer; PDAC, pancreatic ductal adenocarcinoma.

Several studies investigated patient characteristics and perioperative factors and their association to TO achievement (*Fig. 4*).

#### Decreased odds of TO (OR 1 or lower)

Parameters associated with decreased odds of achieving TO in multivariate analysis included Charlson–Deyo co-morbidity index 3 or higher, older age (age more than 70 years), and neoadjuvant chemotherapy^6,24,27^ for patients with PDAC, and an ASA score 3 or higher in patients with any pancreatic disease receiving pancreatoduodenectomy^8^. Age more than 65 years, an ASA score more than 2 (or 3 or higher), the Model for End-Stage Liver Disease score, and an albumin-bilirubin grade 2/3 were independent predictors of TO in patients with hepatic malignancies^33,39^. Metabolic syndrome, ASA score of 3 or higher, and previous hepatic surgery were negatively associated with TO in patients undergoing liver surgery for any liver disease^9,23^.

More than one previous hospital admission was associated with decreased odds of TO (odds decreasing by the increasing number of previous stays) in patients undergoing HPB surgery for any indication^15^.

#### Increased odds of TO (OR more than 1)

Female sex was an independent predictor of TO in patients undergoing HPB surgery both for cancer or any indication^8,15,27,28^. Omission of neoadjuvant chemotherapy in patients undergoing distal pancreatectomy, and a dilated pancreatic duct (more than 3 mm) as well as diagnosis of PDAC in patients receiving pancreatoduodenectomy were associated with higher likelihood of achieving TO^8^. A Barcelona clinic liver cancer stage 0 (*versus* B/C) and an albumin-bilirubin grade 1 (*versus* 2/3) were independent predictors of TO in patients with HCC^38^. In patients with ICC, age 60 years or younger, no preoperative jaundice, and omission of neoadjuvant chemotherapy increased the likelihood of achieving TO^36^.

## Discussion

In this systematic review the median rate of TO achievement was 38 per cent, ranging widely from 15 to 70 per cent. Major obstacles in achieving TO were ‘complications’, ‘prolonged LOS’, and ‘readmission’. While identification of these parameters might be of great benefit to guide improvement initiatives, it is important to note that they are themselves influenced by patient and procedural factors. LOS and readmission not only depend on surgical quality but also on cultural norms and healthcare policies as payment schemes and access to long-term rehabilitation facilities^41^.

Many factors influenced TO achievement. Laparoscopic approach, less-extended surgery (distal pancreatectomy *versus* pancreaticoduodenectomy and minor *versus* major liver resection), high CMI and surgical volume, and higher socioeconomic status all influenced TO positively. Age, co-morbidity status, and neoadjuvant chemotherapy were also reported as independent predictors of TO after multivariable analysis in some studies^8,9,23,33,39^. Indeed, the retrospective fashion of all these studies severely limits the reliability of this data.

Limitations of this review included heterogeneity of definitions of TO that impeded fair comparisons of TO rates between institutions so that meta-analysis was inappropriate. The retrospective nature of the studies and use of administrative data sets from large databases might have led to selection or secular bias and lack of relevant clinical variables and perioperative parameters^12, 15–23^.

TO definition *per se* has some flaws. TO parameter selections must be conducted carefully, and likelihood of achievement should not influence this decision as it would lead to biased high TO rates as in a self-fulfilling prophecy (for example choosing parameters that positively influence laparoscopy in a centre that performs mainly laparoscopic surgery). Aiming to reach high TO rate should not lead to operations being offered only to the ‘best’ candidates and avoiding those at higher risk. To avoid these biases, TO should be used in addition to other quality metrics such as CMI^11,12,20–22,29^. TO does not include a patient perspective. As patient-reported and clinically defined outcomes may well differ, future studies should further investigate this relationship and incorporate patient-reported outcomes in TO definitions^7^.

TO is a promising multidimensional measure reflecting the ideal outcome after surgery but is still heterogeneously defined. A current definition of TO in HPB surgery should include the terms ‘no prolonged LOS’, ‘no complications’, ‘no readmission’, and ‘no deaths’. Under proper risk adjustment, TO in addition to other quality metrics, might become a useful tool to aid clinical decision-making, provide information on surgical quality for the patient, and assist preoperative patient selection.

## Supplementary Material

zrac149_Supplementary_Data

## Data Availability

The data that support the findings of this study are available on request from the corresponding author.
