# Supplementary material for: Textbook outcome in hepato-pancreato-biliary surgery: systematic review
Source: BJS Open. 2022 Nov 30;6(6):zrac149. doi: 10.1093/bjsopen/zrac149 (PMC9710735; doi:10.1093/bjsopen/zrac149)
Supplement: zrac149_Supplementary_Data [file zrac149_supplementary_data.zip › Supplementary_material.docx]

**Textbook outcome in HPB surgery: systematic review**

E. Pretzsch^1^, D. Koliogiannis^1^, J. G. D’Haese^1^, M. Ilmer^1^, M. O. Guba^1^, M. K. Angele^1^, J. Werner^1^, H. Niess^1^

^1^Department of General, Visceral, and Transplant Surgery, Ludwig-Maximilians-University Munich, Munich, Germany

**Corresponding author:**

Hanno Niess, M.D.

Department of General, Visceral, and Transplant Surgery

Ludwig-Maximilians-University Munich

Munich, Germany

[Hanno.niess@med.uni-muenchen.de](mailto:Hanno.niess@med.uni-muenchen.de)

Orcid ID: <https://orcid.org/0000-0002-0981-4049>

Twitter: @hanno_niess

**Supplementary Materials - Index**

| **Supplementary Tables** |  |
| --- | --- |
| Table S1. Textbook outcome and survival | *pag. 2* |

**Supplementary Tables**

**Table S1. Textbook outcome and survival**

| Author (Year, Journal) | Diagnosis | Procedure | Survival parameters |
| --- | --- | --- | --- |
|  |  | **Pancreatic Surgery** |  |
| Aquina et al. (2021, Annals of Surgical Oncology)^34^ | Malignancy | Pancreatic surgery | improvement in overall survival for every 10% increase in adjusted hospital TOO rate (HR, 0.90; 95% CI, 0.88–0.93) (p<0.0001) |
| Heidsma et al. (2020, Journal of Surgical Oncology)^29^ | PNET | Pancreatic surgery | **Disease-free survival:**  TO: HR 0.49 (95%CI 0.32-0.75) |
| Kulshrestha et al. (2021, Journal of Surgical Oncology)^33^ | PDAC | PD | TO: 4.8 month increase in median survival (p <0.001) |
| Sweigert et al. (2020, Journal of Surgical Oncology)^6^ | PDAC | PD | **OS:** TO: 27 months, no TO: 19.8 months (p<0.001)  **Risk of death with TO:** HR 0.73 (95% CI, 0.70‐0.77) |
| Sweigert et al. (2021, Surgery)^28^ | PDAC | PD | **OS:** TO: 26.7 months, no TO: 21.1 months (p<0.001) |
|  |  | **Liver Surgery** |  |
| Azoulay et al. (2021, JHEP Reports)^27^ | HCC (CSPH≥10mmHg) | Liver surgery | **5-year overall survival:** TO: 55%; (p=not specified) |
| Brustia et al. (2021, Surgery)^24^ | ICC | Liver surgery | - |
| Hobeika et al. (a) (2020, JHEP Reports)^26^ | HCC | Liver surgery | **Disease-free survival:**  TO: (HR 0.34 (95%CI 0.19-0.60) |
| Hobeika et al. (2021, The British Journal of Surgery)^25^ | ICC | Liver surgery | - |
| Tsilimigras et al. (a) (2020, HPB)^35^ | HCC | Liver surgery | **5-year overall survival:** TO: 69.6%, no TO: 56.9% (p<0.001.)  **Risk of death with TO:** HR 0.60 (95% CI 0.42–0.85) |
| Tsilimigras et al. (b) (2020, Annals of Surgical Oncology)^31^ | HCC, ICC | Liver surgery | HCC  **Risk of death with TO:** HR 0.63 (95%CI 0.46–0.85)  ICC  **Risk of death with TO:** HR 0.74 (95%CI 0.56–0.97 |
| Author (Year, Journal) | Diagnosis | Procedure | Survival parameters |
|  |  | **Pancreatic Surgery** |  |
| Aquina et al. (2021, Annals of Surgical Oncology)^34^ | Malignancy | Pancreatic surgery | improvement in overall survival for every 10% increase in adjusted hospital TOO rate (HR, 0.90; 95% CI, 0.88–0.93) (p<0.0001) |
| Heidsma et al. (2020, Journal of Surgical Oncology)^29^ | PNET | Pancreatic surgery | **Disease-free survival:**  TO: HR 0.49 (95%CI 0.32-0.75) |
| Kulshrestha et al. (2021, Journal of Surgical Oncology)^33^ | PDAC | PD | TO: 4.8 month increase in median survival (p <0.001) |
| Sweigert et al. (2020, Journal of Surgical Oncology)^6^ | PDAC | PD | **OS:** TO: 27 months, no TO: 19.8 months (p<0.001)  **Risk of death with TO:** HR 0.73 (95% CI, 0.70‐0.77) |
| Sweigert et al. (2021, Surgery)^28^ | PDAC | PD | **OS:** TO: 26.7 months, no TO: 21.1 months (p<0.001) |
|  |  | **Liver Surgery** |  |
| Azoulay et al. (2021, JHEP Reports)^27^ | HCC (CSPH≥10mmHg) | Liver surgery | **5-year overall survival:** TO: 55%; (p= NA) |
| Brustia et al. (2021, Surgery)^24^ | ICC | Liver surgery | - |
| Hobeika et al. (a) (2020, JHEP Reports)^26^ | HCC | Liver surgery | **Disease-free survival:**  TO: (HR 0.34 (95%CI 0.19-0.60) |
| Hobeika et al. (2021, The British Journal of Surgery)^25^ | ICC | Liver surgery | - |
| Tsilimigras et al. (a) (2020, HPB)^35^ | HCC | Liver surgery | **5-year overall survival:** TO: 69.6%, no TO: 56.9% (p<0.001.)  **Risk of death with TO:** HR 0.60 (95% CI 0.42–0.85) |
| Tsilimigras et al. (b) (2020, Annals of Surgical Oncology)^31^ | HCC, ICC | Liver surgery | HCC  **Risk of death with TO:** HR 0.63 (95%CI 0.46–0.85)  ICC  **Risk of death with TO:** HR 0.74 (95%CI 0.56–0.97 |

CSPH: clinically significant portal hypertension, DP: distal pancreatectomy, HCC: hepatocellular carcinoma, ICC: intrahepatic cholangiocellular carcinoma, PD: pancreatoduodenectomy, PNET: pancreatic neuroendocrine tumor
